# Supplementary material for: Synthesis, Structures and Electrochemical Properties of Lithium 1,3,5-Benzenetricarboxylate Complexes
Source: Polymers (Basel). 2019 Jan 12;11(1):126. doi: 10.3390/polym11010126 (PMC6401979; doi:10.3390/polym11010126)
Supplement: Supplementary file 1 [file polymers-11-00126-s001.pdf]

Supporting Information for

Synthesis, Structures and Electrochemical Properties of Lithium 1,3,5-Benzenetricarboxylate Complexes

Pei-Chi Cheng,[a] Bing-Han Li,[a] Feng-Shuen Tseng,[a] Po-Ching Liang,[a] Chia-Her Lin,[*a] and Wei-Ren Liu[*b]

[a]Department of Chemistry, Chung-Yuan Christian University, Chungli 320, Taiwan; [b]Department of Chemical Engineering, Chung-Yuan Christian University, Chungli 320, Taiwan

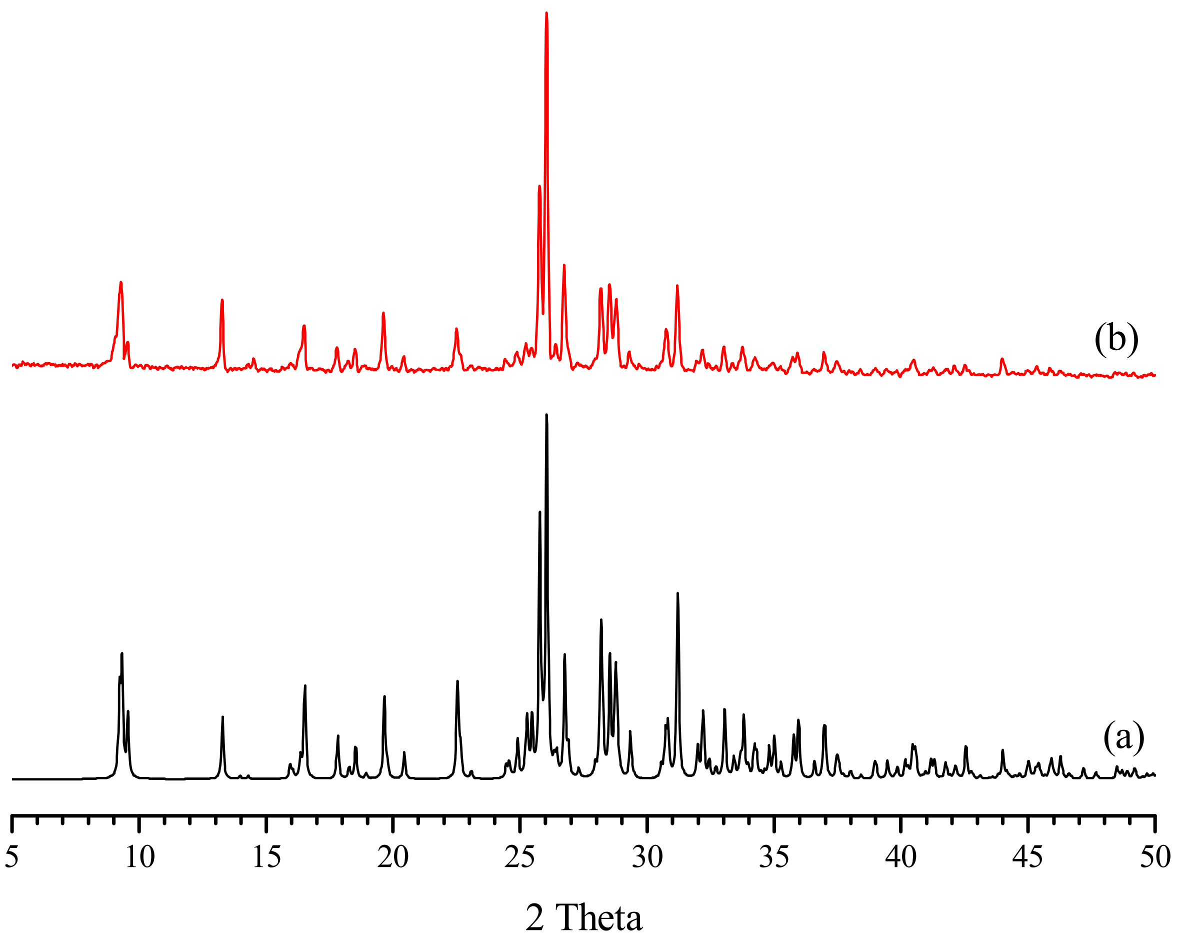

Figure S1. Powder XRD patterns of **1** (**a**) simulated and (**b**) measured.

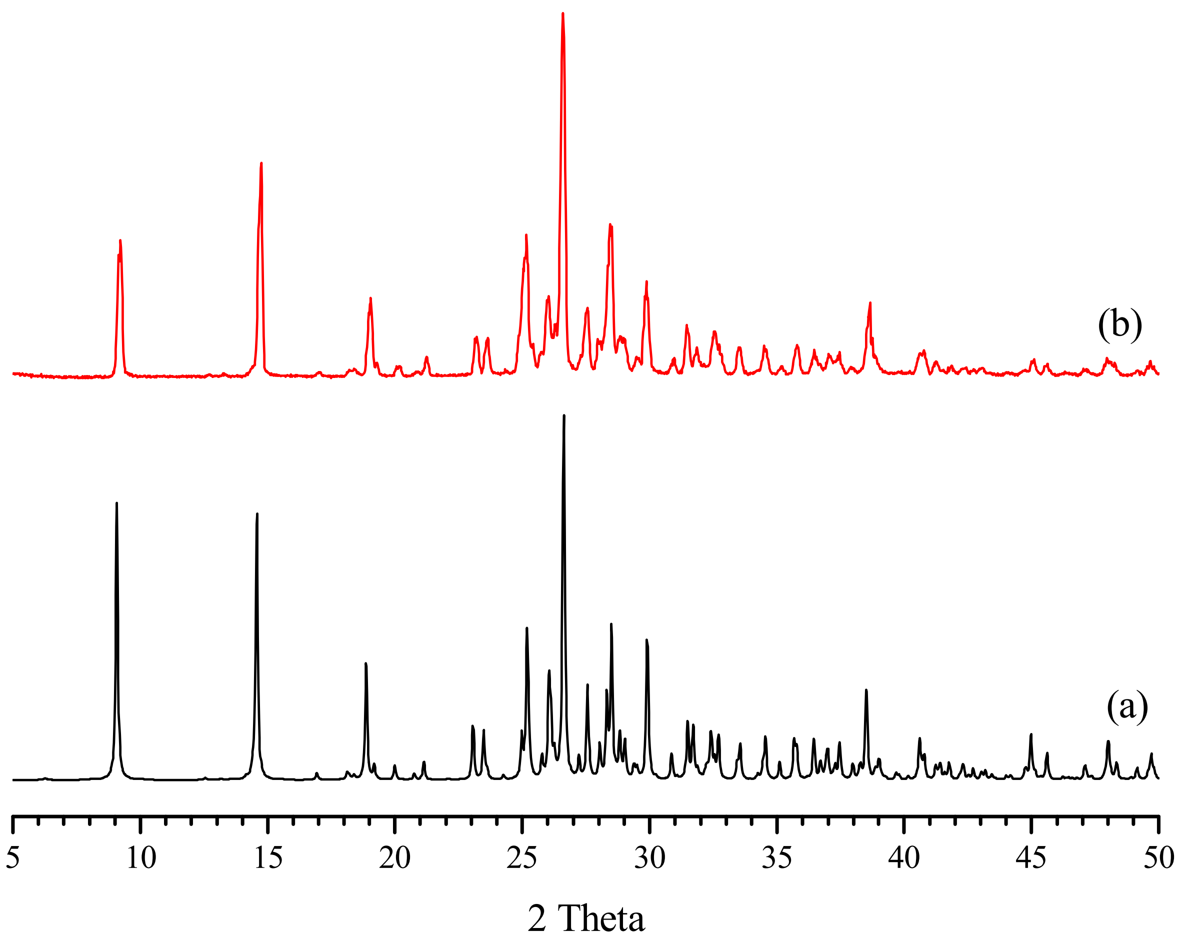

Figure S2. Powder XRD patterns of **2** (**a**) simulated and (**b**) measured.

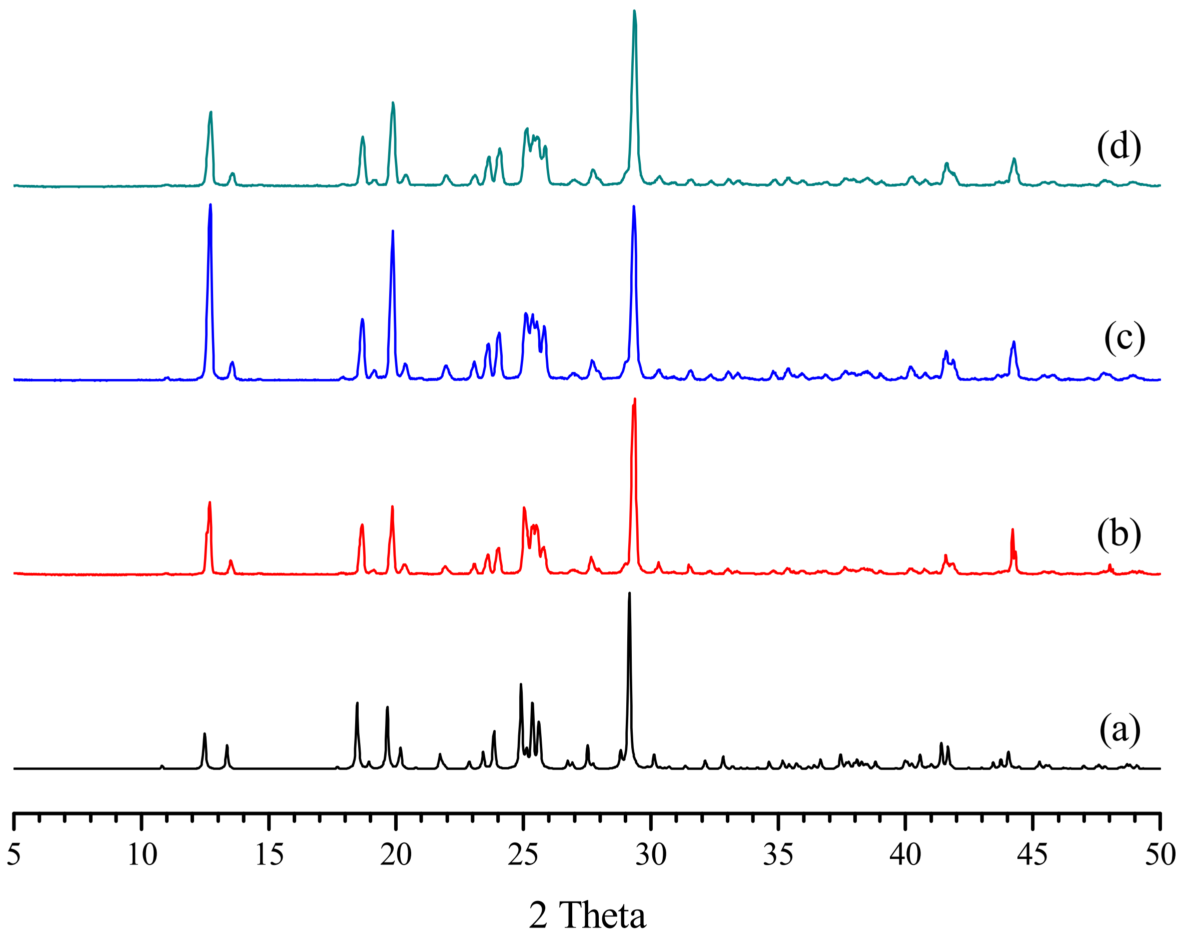

Figure S3. Powder XRD patterns of **3** under different synthesis reaction conditions: (**a**) simulated, (**b**) MeOH/water, (**c**) EtOH/water, (**d**) IPA/water.

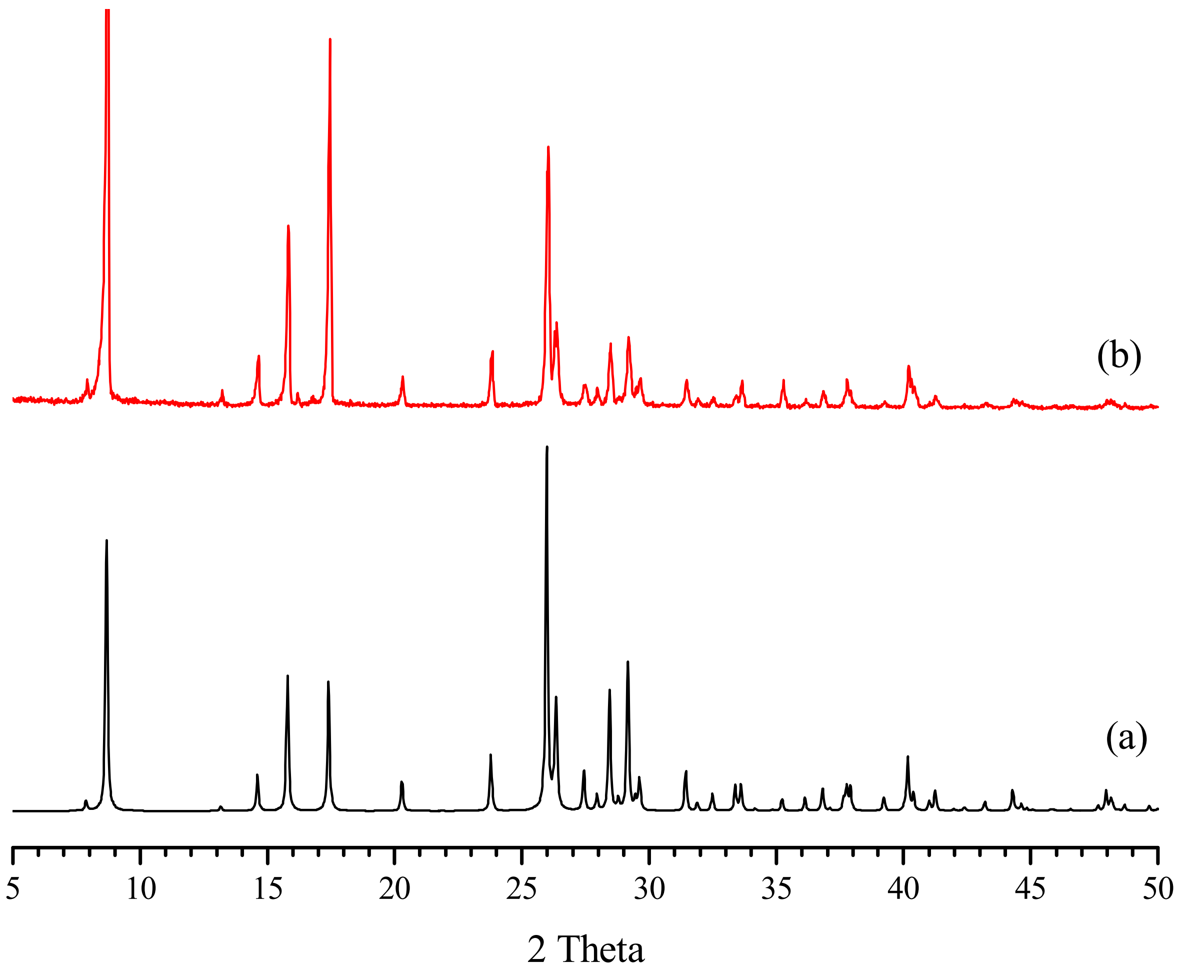

Figure S4. Powder XRD patterns of **4** (**a**) simulated and (**b**) measured.

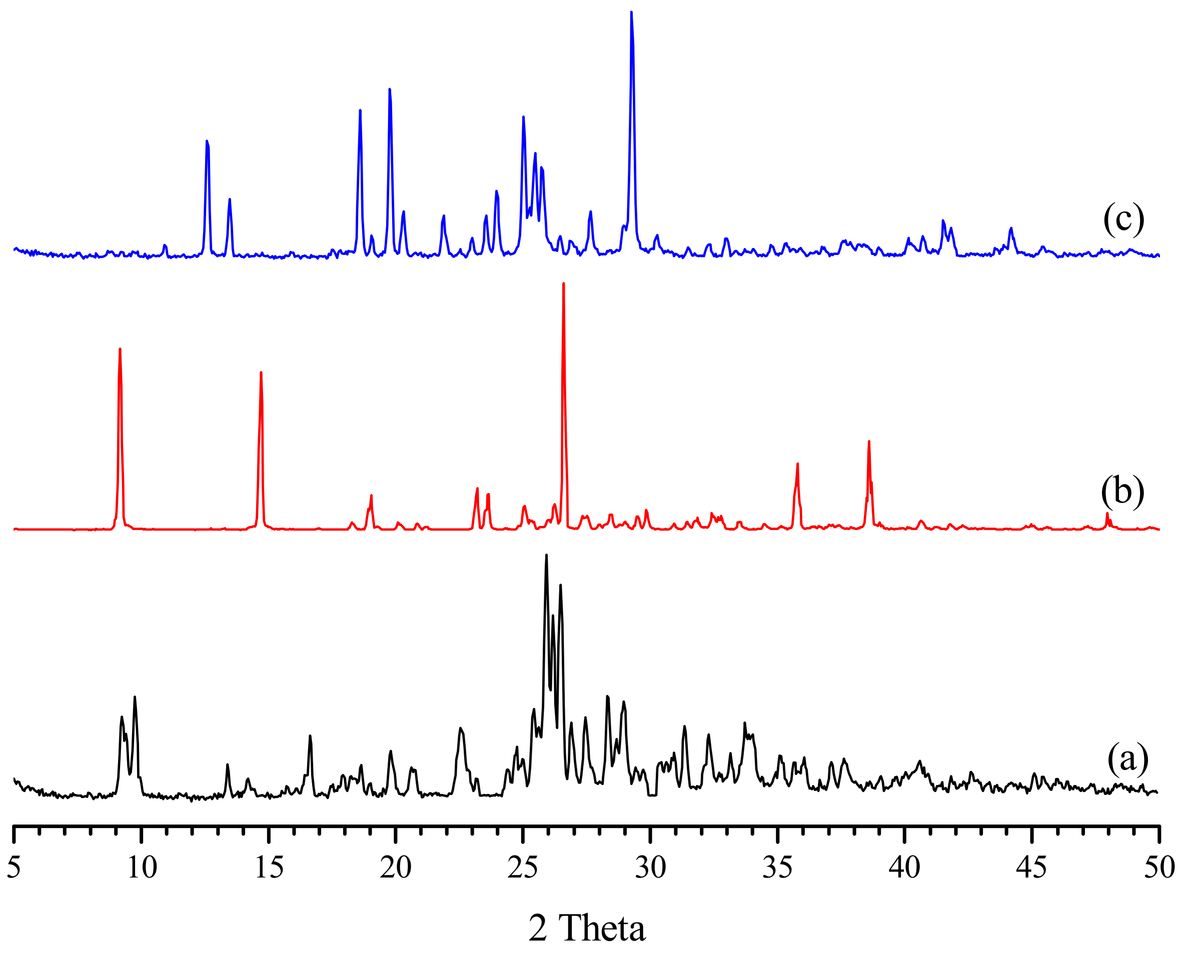

Figure S5. Powder XRD patterns of **1** at (**a**) 30 °C, (**b**) 50 °C, (**c**) 100 °C.

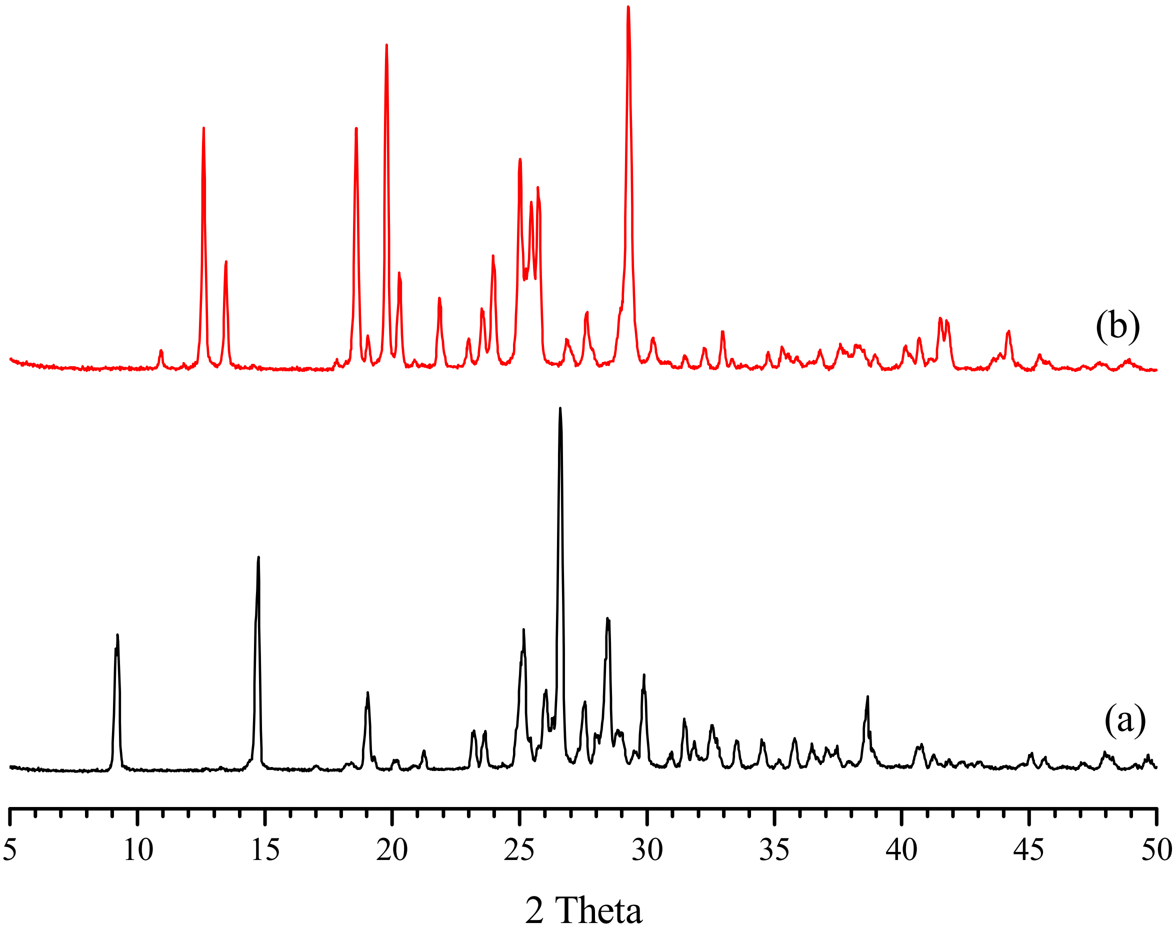

Figure S6. Powder XRD patterns of **2** at (**a**) 30 °C and (**b**) 100 °C.

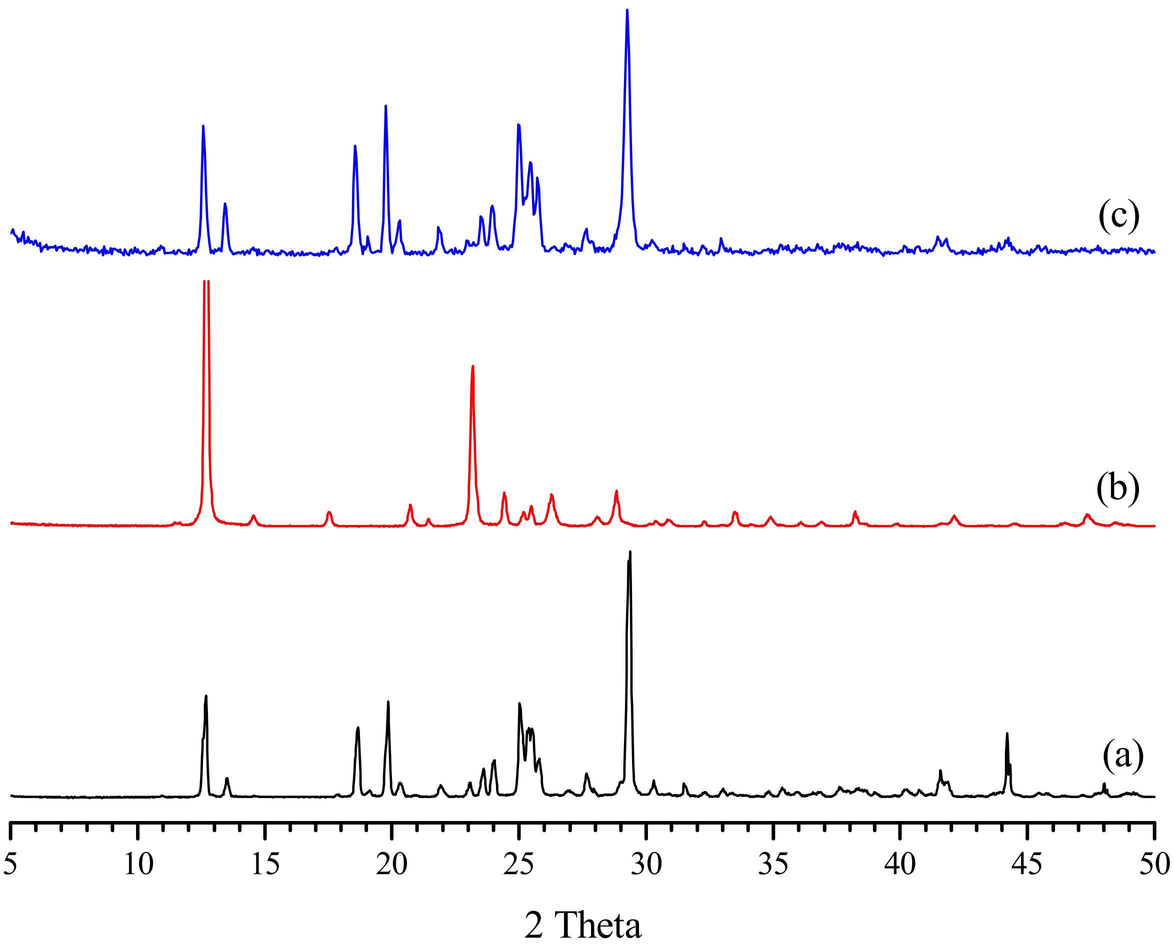

Figure S7. Powder XRD patterns of **3** at (**a**) 30 °C, (**b**) 300 °C, and (**c**) rehydrated species obtained by exposure of the dehydrated species to water for 1 day.

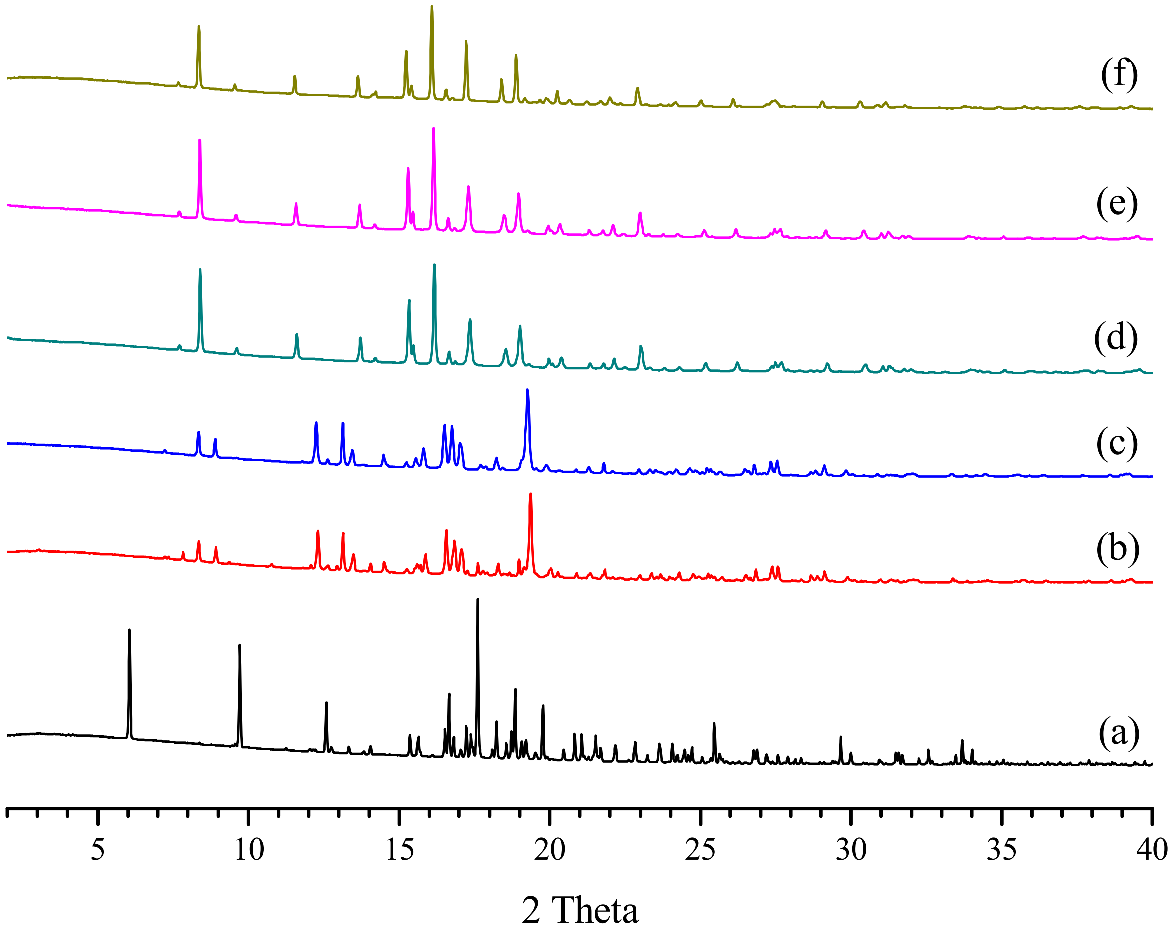

Figure S8. Variable temperature powder XRD patterns (wavelength 1.03321 Å) of **2** at (**a**) room temperature, (**b**) 100 °C, (**c**) 200 °C, (**d**) 300 °C, (**e**) 400 °C, and (**f**) 500 °C.

Table S1. Selected bond lengths (Å) for **1–4**.

		**1**	
Li(1)-O(1)	1.870(3)	Li(2)-O(4W)	1.947(4)
Li(1)-O(1W)	1.991(3)	Li(2)-O(2)#2	2.111(4)
Li(1)-O(2W)	1.856(4)	Li(3)-O(5)	1.905(3)
Li(1)-O(1W)#1	1.978(3)	Li(3)-O(5W)	1.988(3)
Li(2)-O(3)	1.977(3)	Li(3)-O(6W)	1.958(3)
Li(2)-O(3W)	1.975(4)	Li(3)-O(6)#3	1.953(3)
		**2**	
Li(1)-O(1)	1.943(2)	Li(2)-O(11)	2.025(2)
Li(1)-O(2)	1.899(3)	Li(2)-O(5)#2	1.968(3)
Li(1)-O(3)	1.881(3)	Li(3)-O(7)	2.045(3)
Li(1)-O(4)#1	1.922(3)	Li(3)-O(8)	1.869(2)
Li(2)-O(9)	1.967(3)	Li(3)-O(6)#4	2.049(3)
Li(2)-O(10)	1.888(3)	Li(3)-O(11)#3	2.091(3)
		**3**	
Li(1)-O(1)	1.981(2)	Li(2)-O(4)#5	1.892(2)
Li(1)-O(3)#1	2.069(2)	Li(2)-O(7)#6	2.018(2)
Li(1)-O(4)#2	1.962(2)	Li(3)-O(5)	1.886(2)
Li(1)-O(6)#3	1.955(2)	Li(3)-O(7)	2.021(2)
Li(2)-O(6)	1.949(2)	Li(3)-O(2)#7	1.918(2)
Li(2)-O(2)#4	1.934(2)	Li(3)-O(5)#6	2.010(2)
		**4**	
Li(1)-O(1)	1.897(2)	Li(1)-O(1)#1	1.897(2)
Li(1)-O(1W)	2.041(4)	Li(1)-O(1W)#2	2.107(4)

Symmetry transformations used to generate equivalent atoms: For **1**, #1 -x+1,-y,-z+2, #2 -x,-y+1,-z+1, #3 -x,-y+2,-z+1; for **2**, #1 -x+3/2,y+1/2,z, #2 -x+3/2,y-1/2,z, #3 -x+1,-y,-z+1, #4 x-1/2,-y+1/2,-z+1; for **3**, #1 -x,-y,-z+1, #2 x,y,z+1, #3 -x+1,-y+1,-z+2, #4 x+1,y+1,z, #5 x+1,y+1,z+1, #6 -x+1,-y+2,-z+1, #7 -x,-y+1,-z+1; for **4**, #1 x,y,-z+1, #2 x+1,y,z.
